# Outcomes after intra-aortic balloon pump insertion in cardiac surgery patients

**DOI:** 10.5935/0103-507X.20200091

**Published:** 2020

**Authors:** George Samanidis, Georgios Georgiopoulos, Stefanos Bousounis, Panagiotis Zoumpourlis, Konstantinos Perreas

**Affiliations:** 1 Department of Adult Cardiac Surgery, Onassis Cardiac Surgery Center - Athens, Greece.; 2 Department of Cardiology, Hippokration Hospital, University of Athens -Athens, Greece.

**Keywords:** Intra-aortic balloon pump, Heart surgery, Ventricular dysfunction, left, Cardiac output, low, Mortality, Balão intra-aórtico, Cirurgia cardíaca, Disfunção ventricular esquerda, Baixo débito cardíaco, Mortalidade

## Abstract

**Objective:**

To assess whether preoperative versus intraoperative insertion of an intra-aortic balloon pump is associated with lower 30-day mortality or reduced length of hospital stay among patients who had an intra-aortic balloon pump inserted for cardiac surgery.

**Methods:**

This was an observational study of patients who had an intra-aortic balloon pump inserted in the preoperative or intraoperative period of cardiac surgery in our department between 2000 and 2012. We assessed the association between preoperative versus intraoperative insertion of an intra-aortic balloon pump and 30-day mortality in a multivariable logistic regression analysis, including preoperative New York Heart Association class, postoperative atrial fibrillation, reoperation, postoperative creatinine and isolated coronary bypass grafting as cofactors. We used a multivariate linear model to assess whether a preoperative versus intraoperative intra-aortic balloon pump was associated with length of postoperative hospital stay, adjusting for reoperation, isolated coronary bypass grafting, heart valve surgery, sex, age, cardiopulmonary bypass time, aortic cross-clamp time, preoperative patients’ status (elective, urgency or emergency surgery) and preoperative myocardial infarction.

**Results:**

Overall, 7,540 consecutive patients underwent open heart surgery in our department, and an intra-aortic balloon pump was inserted pre- or intraoperatively in 322 (4.2%) patients. The mean age was 67 ± 10.2 years old, the 30-day mortality was 12.7%, and the median length of hospital stay was 9 days (7 - 13). Preoperative versus intraoperative intra-aortic balloon pump insertion did not affect the incidence of 30-day mortality (adjusted OR = 0.69; 95% CI, 0.15 - 3.12; p = 0.63) and length of postoperative hospital stay (β = 5.3; 95%CI, -1.6 to 12.8; p = 0.13).

**Conclusion:**

Preoperative insertion of an intra-aortic balloon pump was not associated with a lower 30-day mortality or reduced length of postoperative hospital stay compared to intraoperative insertion.

## Abstract (pt)

**Objetivo:**

Avaliar se o momento de inserção de um balão intra-aórtico pré-operatório em comparação com o intraoperatório se associa com menor mortalidade em 30 dias ou diminuição do tempo de permanência no hospital entre pacientes que passaram por inserção de um balão intra-aórtico para cirurgia cardíaca.

**Métodos:**

Este foi um estudo observacional de pacientes submetidos à inserção de um balão intra-aórtico no período pré-operatório ou intraoperatório de cirurgia cardíaca em nosso departamento entre 2000 e 2012. Avaliamos a associação entre a inserção pré-operatória em comparação com a intraoperatória de um balão intra-aórtico e a mortalidade em 30 dias em uma análise de regressão logística multivariada, incluindo a classificação pré-operatória segundo a *New York Heart Association*, a presença de fibrilação atrial pós-operatória, a reoperação, a creatinina pós-operatória e a cirurgia de revascularização do miocárdio isolada como cofatores. Utilizamos um modelo linear multivariado para avaliar se a inserção pré-operatória do balão intra-aórtico, em comparação com a intraoperatória, associou-se com o tempo de permanência no hospital após a cirurgia, com ajuste para reoperação, cirurgia de revascularização do miocárdio isolada, cirurgia valvar, sexo, idade, tempo de bypass cardiopulmonar, tempo de oclusão aórtica, condição pré-operatória do paciente (cirurgia eletiva, de urgência ou emergência) e infarto do miocárdio pré-operatório.

**Resultados:**

Foram submetidos à cirurgia cardíaca aberta em nosso departamento 7.540 pacientes consecutivos, tendo sido inserido um balão intra-aórtico em momento pré-operatório ou intraoperatório em 322 (4,2%) pacientes. A média de idade foi 67 ± 10,2 anos, e a mortalidade em 30 dias foi de 12,7%. O tempo mediano de permanência no hospital foi de 9 dias (7 - 13). A inserção pré-operatória de balão intra-aórtico, em comparação com a intraoperatória, não afetou a incidência de mortalidade em 30 dias (RC ajustada = 0,69; IC95% 0,15 - 3,12; p = 0,63) e nem o tempo de permanência no hospital após a cirurgia (β = 5,3; IC95% 1,6 - 12,8; p = 0,13).

**Conclusão:**

Em comparação com a inserção intraoperatória, a inserção pré-operatória de um balão intra-aórtico não se associou com menor mortalidade em 30 dias nem reduziu o tempo de permanência no hospital.

## INTRODUCTION

Intra-aortic balloon pump (IABP) is widely used as a mechanical circulatory support device to prevent and treat low cardiac output syndrome in patients with unstable ischemic heart disease, acute coronary syndrome, heart failure and those who underwent cardiac surgery. Perioperative IABP insertion may improve cardiac output by 10 - 30%, particularly in patients with preoperative low left ventricular ejection fraction (LVEF) and low cardiac output syndrome who are undergoing heart operations.^(1)^

The effectiveness of an IABP lies on its ability to reduce the oxygen demand of the myocardium while facilitating an increase in myocardial oxygen supply. This is achieved through a series of hemodynamic effects, which include increased diastolic pressure and decreased afterload.^(1-3)^ In addition, IABP is indicated in high risk patients with LVEF < 30%, significant left main disease (LMD) with hemodynamic consequences (hemodynamic instability) and acute or chronic heart failure, and patients having difficulty weaning from cardiopulmonary bypass (CPB) during cardiac surgery operations difficulty.^(1,4)^

In this study, we evaluated the risk factors associated with outcomes after IABP insertion in patients who underwent open heart surgery. Our primary aim was to assess whether pre- *versus* intraoperative insertion of IABP is associated with a lower 30-day mortality or smaller length of hospital stay among patients who had IABP inserted in the pre- or intraoperative periods of cardiac surgery.

## METHODS

**Figure 1:**
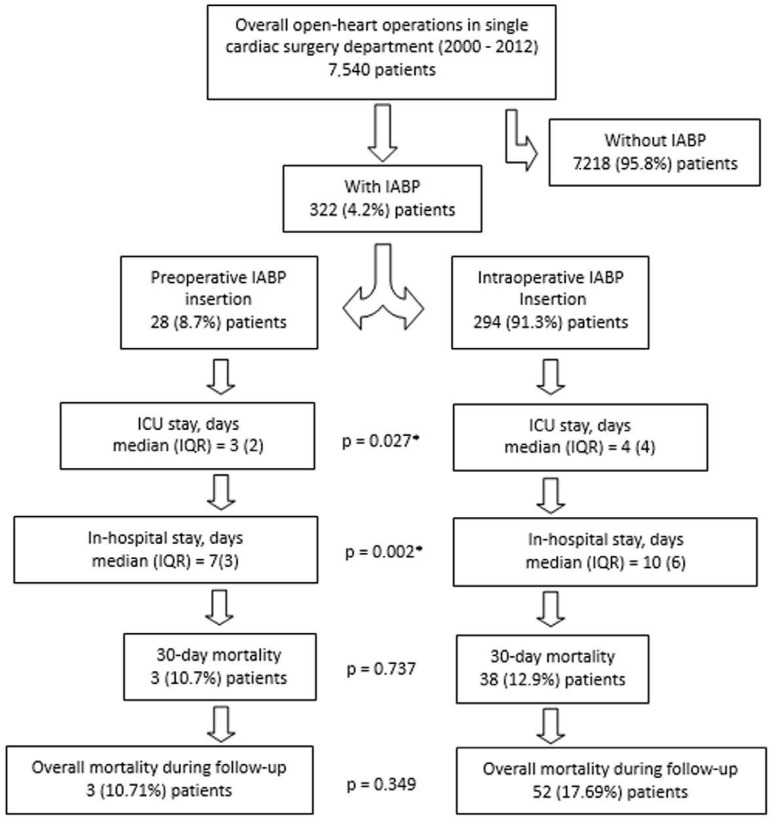
Patients undergoing open heart surgery. IABP - intra-aortic balloon pump; ICU - intensive care unit; IQR - interquartile range.

Between 2000 and 2012 7,540 consecutive patients underwent open heart surgery in our department, and IABP was inserted preoperatively and intraoperatively in 322 (4.2%) patients (Figure 1). All preoperative, intraoperative and postoperative data were recorded in our database. In all patients, IABP insertion was achieved through the left or right common femoral artery (percutaneously).

This study was an observational study including 322 cardiac surgery patients who received IABP (preoperative and intraoperative). Furthermore, this study was approved by the hospital’s institutional ethics committee.

The follow-up was performed in the outpatient clinic, and the first follow-up was 30 days after discharge. The second follow-up was 6 months after the first follow-up date and after one visit/year. When the patients were discharged and did not visit the outpatient clinic, then the 30-day mortality incidence was recorded after telephone contact with the patient (a mandatory process in our hospital). In addition, in our study, the median follow-up of patients was 10 (1 - 49) months.

In this study, we included patients who underwent the entire spectrum of heart operations in our department (excluding off pump coronary bypass grafting (CABG)): isolated CABG, CABG with left descending artery endarterectomy, heart valve replacement, aortic root replacement (modified Bentall operation), ascending aorta replacement and combined heart operations.

### Indications and time of intra-aortic balloon pump insertion

The indications for IABP insertion (in all patients) were: cardiac index < 2.2L/min/m^2^, systolic pressure (SP) < 80mmHg, mean arterial pressure < 50mmHg, intraoperative transesophageal echocardiography of the LVEF < 20 - 25% and severe metabolic acidosis despite the maximal vasoactive/inotropic support (adjusted for each patient and dependent on the body surface area, pulmonary systolic artery pressure, systemic vascular resistant, central venous pressure and cardiac index).

In addition, for analysis purposes, the patients were divided into two groups:

a) Preoperative: in the operating room before patient intubation or in the cardiology intensive care unit (ICU) or catheterization laboratory.
b) Intraoperative: in the operating room after patient intubation and any time before the patient was transferred from the operating room to the ICU.

### Statistical methods

Continuous variables are presented as the mean values ± standard deviation or medians (interquartile range) for non-normally distributed variables. The categorical variables are presented as the absolute values (count) and percentages. Normality of the continuous variables was tested using the Kolmogorov-Smirnov criterion or graphically assessed through P-P plots.

Differences in continuous variables between groups of patients according to the time of insertion of IABP (i.e., mortality after an acute coronary syndrome (ACS) episode at the end of the 10-year follow-up) were evaluated through independent samples Student’s t-test or the nonparametric Mann-Whitney test and chi-squared test for nominal variables.

Subsequently, logistic regression analysis was applied to evaluate the association between baseline predictors and the likelihood of developing 30-day mortality and in-hospital mortality (dependent outcome) after adjusting for various confounders. The primary endpoints were 30-day mortality and survival rate, while secondary endpoints were in-hospital mortality and in-hospital stay. The effect size (i.e., risk of primary endpoint) was reported as the estimated odds ratios (OR) and the corresponding 95% confidence intervals (95%CI). A multivariable linear regression analysis was used to predict the length of in-hospital stay based on the preoperative and intraoperative factors. In addition, Cox proportional-hazards models were used to examine the association between variables of interest and the main endpoints of the study under survival settings. Associations are presented as hazard ratios (HR) with 95%CI. Log rank tests and Nelson-Aalen curves for the cumulative hazard of death across the follow-up were also implemented in the survival analysis. In addition, a competing risk regression analysis was performed to assess the association of baseline predictors with the specific outcome of fatal cardiogenic shock based on Fine-Gray’s proportional subhazard model. In detail, death from cardiogenic shock was flagged as the outcome of interest, while other causes of death (sepsis or multiple organ failure) were set as competing risks. Cumulative incidence curves estimated the probability of this type of death in the setting where other competing risks are acknowledged to exist. Multivariable regression (logistic or survival) models were built under Collett’s approach: all significant univariate (p < 0.05) predictors of death were initially entered in a fully adjusted model and subsequently selected through forward and backward selection loops. Certain variables of biological interest (i.e., age and sex) were included in the final multivariable models. Goodness of fit for logistic regression models was evaluated by the Hosmer-Lemeshow test.

Statistical analysis was performed by STATA package, version 11.1 (StataCorp, College Station, Texas USA). Statistical significance was set at p < 0.05. To avoid overfitting in the final multivariable regression models, a ratio of 5 - 10 events per covariate was retained in all analyses.^(2)^

## RESULTS

### Preoperative details

Of the 322 patients who received IABP, 79.8% were male, with an average age of 67 ± 10.2 years old. Among the patients with dyspnea, most (65.5%) were graded as class I according to a New York Heart Association (NYHA) classification, while most patients (41.3%) with angina were graded as class I according to a Canadian Cardiovascular Society Classification (CCSC). In addition, 40.7% of the patients had a recent (< 90 days) preoperative ACS, and 19.9% suffered from LMD > 50%. The mean preoperative creatinine value was 1.3mg/dL. Furthermore, preoperative LVEF was classified into 3 categories (≤ 35%, > 35 - < 50%, ≥ 50%). The demographic characteristics and preoperative data of the study population are shown in table 1.

**Table 1:** Demographic characteristics

Demographics and preoperative data	
Sex	
Male	257 (79.8)
Female	65 (20.2)
Age (years old)	67 ± 10.2
BSA (m^2^)	1.86 ± 0.19
NYHA	
I	211 (65.5)
II	35 (10.9)
III	13 (4.0)
IV	63 (19.6)
CCSC	
0	84 (26.1)
I	133 (41.3)
II	29 (9)
III	9 (2.8)
IV	67 (20.8)
Preoperative acute coronary syndrome	131 (40.7)
Left main diseases (> 50%)	64 (19.9)
Preoperative creatinine (mg/dL)	1.3 ± 0.9
Preoperative left ventricular ejection fraction (%)	
≤ 35	103 (32)
> 35 - < 50	113 (35)
≥ 50	106 (33)

BSA - body surface area; NYHA - New York Heart Association; CCSC - Canadian Cardiovascular Society. Results expressed as n (%) or mean ± standard deviation.

### Intraoperative details

The perioperative details are presented in table 2. Most of the patients (81.4%) had an elective preoperative status, and 91.3% of the patients received IABP intraoperatively. Overall, 61.5% of the patients underwent isolated CABG, while the other types of operations are listed in table 2. Among the patients who underwent isolated CABG, the average number of grafts was 2. Aortic valve replacement was the most frequent operation in the group of patients with heart valve replacement. Ninety patients underwent combined operations.

**Table 2:** Perioperative details

Perioperative details	
Preoperative patient status	
Elective	262 (81.4)
Urgent	46 (14.3)
Emergency	14 (4.3)
IABP insertion	
Preoperative	28 (8.7)
Intraoperative	294 (91.3)
Redo	30 (9.3)
Type of operation	
Isolated CABG	198 (61.5)
Number of grafts	2 ± 1.1
I	15 (4.7)
II	64 (19.7)
III	104 (32.3)
IV	15 (4.7)
CABG with radial artery	31 (9.6)
CABG with free right internal mammary artery	45 (13.9)
CABG with LAD endarterectomy	11 (3.4)
Isolated heart valve surgery	23 (7.14)
Aortic valve replacement	11 (3.4)
Mitral valve replacement	5 (1.6)
Tricuspid valve replacement	1 (0.3)
Double and triple valve surgery	6 (1.9)
Isolated aortic root replacement (modified Bentall operation)	6 (1.9)
Other operations	5 (1.6)
Ischemic left ventricular rupture repair	1 (0.3)
Pericardiectomy	1 (0.3)
Ascending aorta replacement	1 (0.3)
Iatrogenic right atrium injury repair	1 (0.3)
Acute aortic dissection type A repair	1 (0.3)
Overall combined operation	90 (28.0)
CABG with other procedure	75 (23.3)
Heart valve replacement with other procedure (exclude CABG)	11 (3.4)
CABG with carotid endarterectomy	4 (1.2)
Cardiopulmonary bypass time (minutes)	148 ± 68
Aortic cross clamp time (minutes)	98.9 ± 44.6

IABP - intra-aortic balloon pump; CABG - coronary bypass grafting; LAD - left anterior descending artery. Results expressed as n (%) or mean ± standard deviation.

### Postoperative details and follow-up data

The postoperative details and follow-up data, including the cause of death, are presented in table 3. Overall, 22.4% of patients suffered from postoperative acute kidney injury (AKI) (postoperative AKI was defined as a two-fold increase in postoperative creatinine levels in comparison to preoperative levels), and 23.6% had at least one postoperative episode of paroxysmal atrial fibrillation. Postoperative cerebrovascular accidents and amputation of the right leg were recorded in three and one patients, respectively. The median ICU and in-hospital stay was 4 and 9 days, respectively. In addition, the 30-day and in-hospital (beyond 30 day) mortality were 12.7% and 4.3%, respectively. All cause mortality during the median follow-up of 10 months was observed in 55 patients (17.1%).

**Table 3:** Postoperative details, follow-up data and cause of death

Postoperative details and follow-up data	
Postoperative troponin value (pg/mL)	
Immediate after operation	9.66 (5.2 - 17.1)
Peak value after operation	19.5 (9.1 - 44)
Postoperative maximum creatinine after operation (mg/dL)	1.5 (1.1 - 2.5)
Acute kidney injury	72 (22.4)
Postoperative atrial fibrillation	76 (23.6)
Intensive care unit stay (day)	4 (2 - 6)
In-hospital stay (day)	9 (7 - 13)
30-day mortality	41 (12.7)
Causes of death	
Multiple organ failure	13 (31.7)
Cardiogenic shock	22 (53.7)
Sepsis	6 (14.6)
In-hospital mortality (beyond 30 days)	14 (4.3)
Causes of death	
Multiple organ failure	4 (28.6)
Cardiogenic shock	2 (14.3)
Sepsis	8 (57.1)
Overall mortality	55 (17.1)
Median follow-up (months)	10 (1 - 49)

Results expressed as median (interquartile range) or n (%).).

### Time of intra-aortic balloon pump insertion (preoperative versus intraoperative)

Preoperative IABP insertion was recorded in 28 patients - elective operations with preoperative LVEF < 25% and ischemic heart disease in 18 patients, emergency operations with ACS and cardiogenic shock in 3 patients, emergency operations after unable percutaneous coronary intervention (PCI) in catheterization laboratory with ACS and hemodynamic instability in two patients, emergency operations for mechanical complications due to ACS in two patients (interventricular septum rupture and free wall of the left ventricular rupture), emergency operation with left main thrombosis and hemodynamic instability in one patient, emergency operation after unable PCI in catheterization laboratory without ACS and hemodynamic instability in 1 patient and preoperative cardiac arrest in the operating room in one patient.

When the study population was divided into two subgroups based on the time of insertion of IABP, the patients with intraoperative treatment with IABP were classified in the more severe class of angina (CCSC IV) and were more likely to be treated with an emergency surgery (Table 4). In addition, the preoperative IABP group presented with smaller increases in postoperative creatinine, decreased aortic cross clamp (ACC) and CPB time and decreased length of in-hospital and ICU stays compared to the intraoperative IABP group (Table 4).

**Table 4:** Comparison between patients with preoperative and intraoperative intra-aortic balloon pump insertion in terms of baseline and preoperative and postoperative characteristics

Variable	Preoperative IABP insertion n = 28	Intraoperative IABP insertion n = 294	p value
Age (years)	64 (11.1)	67.3 (10.1)	0.142
Sex, male	7 (25.0)	58 (19.73)	0.507
NYHA class IV	4 (16.0)	59 (22.18)	0.823
CCSC class IV	14 (56)	53 (19.9)	0.003*
Left main disease	6 (21.43)	64 (21.77)	0.967
Acute kidney injury	3 (10.71)	69 (23.55)	0.280
Left ventricular ejection fraction ≤ 35%	12 (42.86)	91 (33.58)	0.604
Redo surgery	1 (3.57)	41 (13.95)	0.119
Isolated coronary artery bypass grafting	21 (75.0)	177 (60.20)	0.124
Heart valve surgery	0 (0)	23 (7.82)	0.125
Combined operation	5 (17.86)	85 (28.91)	0.213
Postoperative atrial fibrillation	4 (14.29)	72 (24.49)	0.224
Preoperative elective status	16 (57.14)	244 (82.99)	0.001*
Preoperative emergency status	7 (25.0)	8 (2.72)	< 0.001*
Preoperative creatinine (mg/dL)	0.9 (0.45)	1.1 (0.5)	0.018*
Peak postoperative creatinine (mg/dL)	1.25 (0.85)	1.5 (1.4)	0.023*
Troponin after surgery (pg/mL)	7.19 (9.2)	9.89 (12.2)	0.042*
Peak troponin after surgery (pg/mL)	12.8 (22.9)	19.9 (36.9)	0.113
CABG number of grafts	2 (1)	2 (2)	0.65
Aortic cross clamp time (minutes)	72 (53)	96 (55)	0.023*
Cardiopulmonary bypass time (minutes)	110 (55)	135 (75)	0.001*
Intensive care unit stay (days)	3 (2)	4 (4)	0.027*
In-hospital stay (days)	7 (3)	10 (6)	0.002*
30-day mortality	3 (10.71)	38 (12.93)	0.737
In-hospital death beyond 30 days	0 (0)	14 (4.76)	0.238
Overall mortality	3 (10.71)	52 (17.69)	0.349

IABP- intra-aortic balloon pump; NYHA - New York Heart Association; CCSC- Canadian Cardiovascular Society; CABG - coronary bypass grafting. p-value is derived from independent samples t-test for continuous variables or Mann-Whitney test and Chi-squared test for nominal variables.

* Statistical significance was set at p < 0.05. Results expressed as n (%) or median (interquartile range).

Interestingly, preoperative insertion of IABP was favorably associated with in-hospital stay (p = 0.02). In addition, a multivariable linear regression analysis was used to calculate the possible effect of the time of IABP insertion on the length of in-hospital stay. After adjusting for reoperation, isolated CABG, heart valve surgery, combined operation, sex, age, CPB time, ACC time, preoperative patients’ status, LVEF and preoperative myocardial infarction (MI), the time of IABP insertion did not predict the length of in-hospital stay (β = 5.3; p = 0.13; 95%CI -1.58 - 12.8).

### 30-day mortality and in-hospital mortality

After univariable logistic regression analysis and among variables of interest, sex (p = 0.006), NYHA class (p < 0.001), postop creatinine (p = 0.004), postop maximum troponin level (p = 0.004), heart valve surgery (p = 0.012), modified Bentall operation (p = 0.016), redo surgery (p < 0.001), ACC (p = 0.006), postop atrial fibrillation (p < 0.001) and isolated CABG instead of combined operation (p < 0.001) correlated with the 30-day mortality, while age (p =0.066), preop ACS (p = 0.052) and LMD (p = 0.055) were not associated with 30-day mortality. After multi-adjustment, only NYHA class, postoperative atrial fibrillation, redo surgery, postoperative creatinine and isolated CABG determined the 30-day mortality, while the time of IABP insertion did not affect the incidence of 30-day mortality (Table 5). Preoperative status of the patients (elective, urgent or emergent) was not associated with overall mortality across the follow up of our study (p = 0.863).

**Table 5:** Results from multiadjusted logistic regression models that evaluated risk factors for 30-day mortality

Risk factors	30-day mortality		
	OR	95%CI	p value
Time of IABP insertion (preoperative versus intraoperative)	0.69	0.15 - 3.12	0.63
Age	1.024	0.975 - 1.076	0.34
Sex	1.21	0.438 - 3.32	0.71
Preoperative NYHA	1.46	1.1 - 1.95	0.01*
Reoperation	4.58	1.43 - 14.7	0.01*
Isolated coronary bypass grafting	0.155	0.052 - 0.460	0.001*
Isolated heart valve surgery	0.271	0.062 - 1.18	0.08
Postoperative creatinine	1.24	1.001 - 1.52	0.04*
Postoperative atrial fibrillation	0.092	0.011 - 0.783	0.02*

OR - odds ratio; 95%CI - 95% confidence interval; IABP - intra aortic balloon pump; NYHA - New York Heart Association. Risk factors in the final multivariable logistic regression models for outcome (30-day mortality) were selected by Collett's method as described in the statistical analysis.

* Statistical significance was set at p < 0.05.

When deaths from cardiogenic shock (n = 24) were assessed individually, only 3 parameters determined the incidence of this outcome, independently of age and sex: NYHA class (OR = 1.51; p = 0.011), type of surgery (isolated CABG *versus* combined surgery) (OR = 0.10; p = 0.004) and the ACC time (OR = 1.01; p = 0.047).

### Survival analysis

**Figure 2:**
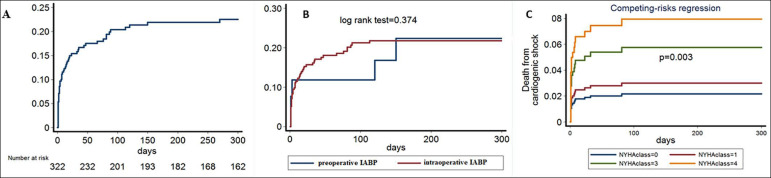
Survival analyses. (A) Nelson-Aalen cumulative hazard estimates for the incidence of death across the follow up of our study. (B) Nelson Aalen curves for the cumulative incidence of death in our cohort according to the time of insertion of intra-aortic balloon pump. (C) Survival analyses. Cumulative incidence for death from cardiogenic shock according to the New York Heart Association classification of patients under competing risk regression analysis. IABP - intra-aortic balloon pump; NYHA - New York Heart Association.

During the median follow-up of 10 months, 55 deaths were recorded (Figure 2A). Among numerous baseline parameters, the final multivariable model for death prediction included age (HR = 1.05; p = 0.009), sex (HR = 0.832; p = 0.606), NYHA class (HR = 1.36; p = 0.002), postop maximum creatinine (HR = 1.26; p < 0.001), postop maximum troponin (HR = 1.002; p = 0.024) and isolated CABG *versus* combined operation (HR = 0.213; p < 0.001). Time of insertion of IABP was not associated with the survival outcome in our study (log rank test, p = 0.374) (Figure 2B).

Upon completing risk regression analysis, only the performance of isolated CABG instead of combined surgery (HR = 0.113; p = 0.003) and NYHA class (HR = 1.41; p = 0.008) (Figure 2C) predicted death from cardiogenic shock independently of age, sex and all baseline confounders. The time of insertion of IABP was not a significant predictor (HR = 0.654; p = 0.487) of death from cardiogenic shock in competing risk analysis.

## DISCUSSION

In recent years, many circulatory support devices have been proposed for hemodynamic support in critically ill patients in cardiology, ICU and cardiac surgery departments. Currently, extracorporeal membrane oxygenation (ECMO), percutaneous mechanical circulatory support, left ventricular assist devices, biventricular assist devices and IABP are the most common devices for circulatory support, particularly in cardiogenic shock due acute or chronic heart disease. On the other hand, many serious and fatal complications are observed during and after placement of these devices. Intra-aortic balloon pump compared to other devices has advantages because of its placement, checking of the correct position and easier management. For these reasons, IABP can be implemented in hospitals and departments without cardiac surgery support. In addition, IABP remains a useful device in critically ill cardiac surgery patients with perioperative low cardiac output syndrome who undergo elective or emergency cardiac surgery operations. The mortality rate in cardiac surgery patients who have received IABP is high, ranging from 10 - 40%, and it is affected by decision making with regard to which patients should be placed on IABP and when, with minimum adverse effects on patient outcomes (morbidity and mortality).^(5-10)^ Intra-aortic balloon pump placement is an additional risk factor in already critically ill cardiac surgery patients. Malperfusion syndrome, limb ischemia, cerebrovascular accidents due to peripheral vascular disease, descending and abdominal aorta calcification and IABP malposition or displacement are the most common complications after IABP insertion.

The most discussed issues with regard to IABP in cardiac surgery remain the time of insertion (preoperative, intraoperative and postoperative), age of cardiac surgery patients and type of heart operation.

As seen from many recent studies, preoperative IABP insertion has many benefits with regard to in-hospital mortality and postoperative adverse cardiac events.^(6-9)^ Our study had comparable results with these studies, and we concluded that preoperative IABP insertion decreased in-hospital stay (p = 0.028) in our study population but did not influence in-hospital mortality (p = 0.374). Poirier et al. analyzed 46,067 patients and concluded that the preoperative insertion of IABP presented a decrease of in-hospital mortality and ICU stay, while its intraoperative and postoperative use was not clearly associated with clinical benefits.^(5,6)^Another recent review (9,212 patients) by Deppe et al. supports the beneficial effect of preoperative IABP insertion in high risk patients before CABG, with decreased risk of mortality, myocardial infarction, renal failure, hospital and ICU stay.^(7)^In 2010, a study with 7,440 patients concluded that in-hospital and risk-adjusted mortality were lower when IABP was used before (10%) than during (16%) or after (29%) the cardiac surgery.^(8)^This was also supported by another study in the same year, which showed that patients with cardiogenic shock complicating acute myocardial infarction who underwent PCI assisted by IABP had a more favorable in-hospital outcome and lower in-hospital mortality than patients who received IABP after PCI.^(9)^However, a study with 173 patients published in 2013 suggested that postoperative IABP use is more promising, showing that the cumulative 30-day mortality was 44% in the group with preoperative IABP of patients and 37% in the postoperative IABP group.^(10)^ In contrast to the aforementioned studies, a recent study in 2014 concluded that IABP treatment commenced before or after PCI was not an independent predictor of mortality.^(11)^ On the other hand, cardiac surgery patients are different than patients who undergo PCI; in cardiac surgery, we have prognostic scores in our armamentarium for worse outcomes (predicted mortality rates by the European System for Cardiac Operative Risk Evaluation (Euroscore) and the Society of Thoracic Surgeons (STS) score. Perhaps preoperative IABP insertion should be considered as a predictive factor in the Euroscore II or STS score for evaluating the possible benefits of preoperative IABP placement in critically ill patients.

Another major risk factor that seems to be associated with 30-day and in-hospital mortality of patients with IABP is the age of the patient. Most studies have already found that older patients have higher mortality rates. These incidents are not surprising because the number of older patients is growing, and they have more coexisting diseases (such as chronic kidney injury, peripheral vascular disease and others), which affect outcomes of critically ill patients, particularly cardiac surgery patients (independent of the need for IABP-support).^(12,13)^ More specifically, a study in 2016 with 522 patients found that age above 70 years was an independent risk factor for mortality within 30 days.^(12)^ In addition, in 2015, another working group of 572 patients concluded that patients older than 65 years undergoing IABP support had higher in-hospital mortality rates in comparison to younger patients.^(14)^In the same direction, two studies in 2015 and 2016 included advanced age as one of the most important predictors of in-hospital mortality and adverse long term prognosis.^(15,16)^Finally, a study with 134 elderly patients showed that patients > 80 years had higher mortality rates than those < 80 years after IABP placement.^(17)^ After multivariate analysis in our study, we showed that age of the patients was a risk factor for survival after IABP insertion (p = 0.009). Of note, the number of older patients with comorbidities undergoing cardiac surgery operations has increased in recent years, and this directly affects outcomes of patients who have received IABP.

Currently, little data have been published about the types of cardiac operations (with IABP placement) as risk factors for 30-day and in-hospital mortality. In our study, we compared the mortality rates between isolated CABG, combined or valve replacement surgical procedures. In 2016, a study with 522 patients suggested that CABG combined with valve surgery should be identified as an independent risk factor for 30-day mortality. Among those who had that type of surgery, 47.5% died within 30 days; among those who did not have such a procedure, the mortality rate was 26.3%.^(12)^ In 2010, another study with 136 patients requiring an IABP concluded that the “operation-specific” mortality was higher in the heart valve surgery population. More specifically, the mortality rates were 21.2%, 50% and 64.3% among those who had isolated CABG, CABG and valve surgery and isolated valve surgery, respectively.^(18)^ In our study, after multiadjusted logistic regression analysis, we found that isolated CABG was a risk factor for 30-day mortality (p = 0.001), and it was possible that most of these patients underwent an emergency CABG operation.

### Limitations of the study

This study included a small number of patients from a single department. Furthermore, the number of patients who received IABP preoperatively (compared with intraoperative) was too small to make safe conclusions. Long-term follow-up is required to demonstrate the potential benefits of preoperative or intraoperative IABP insertion in cardiac surgery patients who are already at high preoperative and intraoperative risk.

## CONCLUSION

Overall, 30-day mortality is substantial in patients needing intra-aortic balloon pump insertion in the pre- or intraoperative periods of cardiac surgery. Preoperative insertion of an intra-aortic balloon pump was not associated with lower 30-day mortality or lower length of postoperative hospital stay compared to intraoperative insertion after adjusting for potential confounders.
